# Non-Invasive Assessment of Liver Fibrosis in Hepatitis B Patients

**DOI:** 10.3390/jcm13041046

**Published:** 2024-02-12

**Authors:** Chinmay Bera, Nashla Hamdan-Perez, Keyur Patel

**Affiliations:** Division of Gastroenterology, University Health Network Toronto, Toronto General Hospital, 200 Elizabeth Street, Toronto, ON M5G 2C4, Canada; nashla.hamdan@uhn.ca (N.H.-P.);

**Keywords:** chronic hepatitis B, non-invasive tests, elastography, fibrosis, APRI, FIB-4

## Abstract

The aim of this review is to provide updated information on the clinical use of non-invasive serum and imaging-based tests for fibrosis assessment in chronic hepatitis B (CHB) virus infection. In recent years, non-invasive tests (NIT) have been increasingly used to determine eligibility for treatment. Liver biopsy is still considered the gold standard for assessing inflammatory activity and fibrosis staging, but it is an invasive procedure with inherent limitations. Simple serum markers such as APRI and FIB-4 are limited by indeterminate results but remain useful initial tests for fibrosis severity if imaging elastography is not available. Point-of-care US-based elastography techniques, such as vibration-controlled transient elastography or 2D shear wave elastography, are increasingly available and have better accuracy than simple serum tests for advanced fibrosis or cirrhosis, although stiffness cut-offs are variable based on E-antigen status and inflammatory activity. Current NITs have poor diagnostic performance for following changes in fibrosis with antiviral therapy. However, NITs may have greater clinical utility for determining prognosis in patients with CHB that have advanced disease, especially for the development of hepatocellular carcinoma and/or liver decompensation. Algorithms combining serum and imaging NITs appear promising for advanced fibrosis and prognostic risk stratification.

## 1. Introduction:

The most current estimate from the World Health Organization (WHO) places the global seroprevalence of hepatitis B virus (HBV) infection at 3.8%; in 2019, it was estimated that 296 million individuals were infected with chronic hepatitis B (CHB), with HBV-associated mortality of 820,000 [1]. Fibrosis is the wound-healing response to injury that occurs in patients with CHB due to ongoing inflammation with subsequent scar formation. Between 8–20% of patients with untreated CHB have progression to cirrhosis following the diagnosis of liver fibrosis [2]. CHB is a leading etiology of cirrhosis and hepatocellular carcinoma (HCC) in approximately 15–40% of untreated patients [3]. CHB is the most common cause of HCC in Asia and Africa, with global cancer-related mortality attributed to CHB estimated at 192,000 in 2019 [4]. Progression to end-stage liver disease occurs in 8–20% of untreated patients and is associated with a 20% cumulative 5-year risk of decompensation [5]. Thus, an accurate assessment of liver fibrosis severity is important to guide treatment decisions and surveillance in patients living with Hepatitis B. Routine liver tests such as alanine aminotransferase (ALT) or HBV DNA levels are useful for disease activity and provide thresholds for treatment decisions but are inadequate for fibrosis severity. Prior studies have indicated that patients with CHIB that have persistently normal or intermittently elevated ALT and baseline DNA >10,000 copies/mL may have significant fibrosis on liver biopsy [6]. Although variable biopsy selection criteria and definitions of upper limits of normal ALT have been used across studies, one-third of patients with persistently normal ALT and elevated HBV DNA may have significant fibrosis (F2–4) [7].

Liver biopsy is the reference standard for assessment of fibrosis and necro-inflammatory activity, but it is an invasive procedure that is costly, associated with morbidity and mortality, and is liable to sampling error due to heterogeneity in fibrosis distribution and interpretation [8]. Several blood and imaging-based non-invasive tests (NITs) are presently available as diagnostic methods for the assessment of significant fibrosis. Currently, available NITs for the staging of fibrosis incorporate biological (serum biomarkers), physical (imaging assessment of tissue stiffness), or physiological (breath test) methods. An important consideration in the use of NITs is that management decisions for CHB consider not only disease severity but also HBV DNA, liver aminotransferases, and HBV e-antigen status, among other variables. The variable natural history, immune activity, and inflammatory flares in CHB all affect the reliability of current NITs for staging fibrosis. We provide an overview of available NITs with validated data of clinical relevance in patients with CHB [9]. Discussion of NIT in other chronic liver diseases has been discussed elsewhere [10]. 

## 2. Serum Biomarkers:

Available serum biomarkers for liver fibrosis assessment include indirect markers that are relatively “simple” biochemical tests or direct markers that are mostly “complex” proteins derived from myofibroblasts and extracellular matrix remodeling (Table 1). 

### 2.1. AST-to-Platelet Ratio Index

AST-to-platelet ratio index (APRI) was initially developed and validated for significant fibrosis and cirrhosis in chronic hepatitis C (CHC) infection. Its primary benefit is that it is cheap and readily available as the algorithm is based on routine blood tests performed in liver clinics. Index thresholds of <0.5 and >1.5 are used to “rule-out” or “rule-in” significant fibrosis (F2–4), and <1 and >2 are respective thresholds for cirrhosis. However, 30–40% of patients have non-diagnostic or “indeterminate” scores between these cut-offs. Another meta-analysis that included 17 studies (*n* = 3573) and 11 studies (*n* = 2083) assessed the effectiveness and accuracy of APRI for predicting CHB-related significant fibrosis (METAVIR F2-F4) and cirrhosis (METAVIR F4), respectively [11]. For significant fibrosis, using variable optimal APRI thresholds, the AUROC ranged from 0.61 to 0.86, and the Summary AUROC(SROC) was 0.77. For assessing the presence of cirrhosis, the SROC was 0.75 (range 0.50–0.83), and pooled sensitivity and specificity in 10 studies were 61% and 75%, respectively. Another meta-analysis that included 34 studies (*n* = 8855) indicated that at cut-off thresholds of 0.5 and 1.5, the summary sensitivities and specificities were 70.0% and 60.0% for significant fibrosis. For cirrhosis (34 studies, *n* = 8773), the SROC was 0.73 [12].

There was significant heterogeneity across studies with different APRI thresholds resulting in modest performance for significant fibrosis and cirrhosis. Indeterminate scores for APRI further reduce clinical utility. However, despite these limitations, APRI may still have a role in the initial estimation of disease severity, especially in resource-limited areas. However, results should be carefully interpreted based on the context of use and phase of CHB infection or hepatitis.

### 2.2. FIB-4

This simple marker was originally developed to identify advanced fibrosis (F3–4) in patients coinfected with human immunodeficiency virus (HIV) and hepatitis C virus (HCV) [13]. FIB-4 includes serum AST, ALT, Platelet count, and age (Table 1). Index thresholds of <1.45 and >3.25 “rule-out” or “rule-in” advanced fibrosis and 30–40% of patients have indeterminate scores of 1.45–3.25. FIB-4 has been validated in different CHB cohorts. In a study that included 1168 Chinese patients with HBV, FIB-4 performance was assessed for advanced fibrosis (F3 and F4) with a cut-off value of 1.433–1.858, respectively, showing a sensitivity of 94%, specificity of 46%, and AUROC of 0.79 [14]. A meta-analysis included 12 studies with 1908 patients with CHB that had significant fibrosis and 10 studies including 2105 patients with CHB that had cirrhosis. The performance of FIB-4 for identifying advanced fibrosis using the cut-off values of <1.45 and >1.62 had a sensitivity of 78% and specificity of 65% with SROC 0.89. The recommended cut-off values for identifying patients with cirrhosis were between 2.9 and 3.6 with an AUROC of 0.96, sensitivity of 42%, and specificity of 96%. These data showed that (as with all NITs) the selection of cut-offs can be used to optimize sensitivity or specificity. Newer test cut-offs derived from heterogenous CHB cohorts, with varying prevalence of fibrosis severity and CHB infection phase, still require validation [15]. In general, upper thresholds of FIB-4 have good specificity in identifying patients with CHB that have advanced fibrosis and cirrhosis. A retrospective study that included 3096 patients with CHB, that had available biopsies from eight clinical trials and two tertiary referral center cohorts, indicated APRI and FIB-4 at conventional cut-offs were associated with misclassification rates for cirrhosis of 41–45%. A newer FIB-4 threshold of ≤0.70 was proposed to exclude cirrhosis with a high sensitivity of 91% in patients older than 30 years of age [16]. As with APRI, optimal cut-offs for fibrosis severity are yet to be determined for different phases of CHB infection or hepatitis.

### 2.3. FibroTest

FibroTest (FT) is a proprietary test and one of the most validated blood-based tests for chronic liver disease. It was originally developed for patients with chronic hepatitis C. FibroTest^®^ (BioPredictive, Paris, France; FibroSURE, LabCorp, Burlington, NC, USA) combines five serum biomarkers that have been associated with liver fibrosis (alpha-2macroglobulin (A2M), apolipoprotein A1, haptoglobin, γ-glutamyl transpeptidase and bilirubin [17,18]. FT provides an index score without indeterminate results, but as this test panel includes analytes such as bilirubin and other acute phase reactants, false results may be observed with Gilberts, haemolysis, cholestasis, or acute inflammatory states. Proprietary tests are limited by cost and availability, especially in resource-limited areas. Thus, simple tests such as APRI and FIB-4 are often used for initial assessment. A meta-analysis from the test manufacturer that included 4 studies (*n* = 322 CHB) evaluated its accuracy for identifying significant fibrosis, with an AUROC of 0.83 [19]. Another meta-analysis that included 11 studies with 1640 patients showed that the SROC for detecting significant fibrosis and cirrhosis was 0.84 and 0.90, respectively [11]. Another meta-analysis of FT included 16 studies (*n* = 2494 CHB) for F2–4 and 13 studies (*n* = 1754) for cirrhosis. A validated FT cut-off of 0.48 was associated with sensitivity and specificity of 61% and 80% for F2–4; an FT cut-off of 0.74 was associated with sensitivity and specificity of 62% and 91% for CHB-related cirrhosis. As with other meta-analyses in CHB, study heterogeneity, variable optimal test thresholds, and CHB cohort differences limit interpretation, but FT appeared to have better diagnostic utility for cirrhosis than significant fibrosis.

### 2.4. Forns Index

This simple marker algorithm was developed in CHC for significant fibrosis and combines age, GGT, cholesterol, and platelet count (Table 1) [20]. In a small study that validated the score in 78 patients with CHB using a cut-off point of ≥4.05, the AUROC was 0.680, with a sensitivity of 75.6 and a specificity of 59.4 to identify patients with significant fibrosis [21].

### 2.5. Hui Score

This score was developed to identify patients with CHB without significant fibrosis in 333 patients with CHB, who were viremic and treatment naïve. Hui’s model uses body mass index (BMI), platelets, bilirubin, and albumin; using the low cut-off value of <0.15, the presence of significant fibrosis could be ruled out with an AUROC 0.791 and an NPV of 0.92 [22].

### 2.6. Other Markers

There is limited data for other direct markers in CHB cohorts for Fibrosis assessment. In a study that included 255 patients with CHB, evaluated FT, Fibrometer^TM^ (Echosens, Paris, France), and Hepascore (PathWest, Perth, WA, Australia), the AUROC ranged from 0.75–0.84 for significant fibrosis, 0.82–0.85 for F3–4, and 0.84–0.87 for cirrhosis, with no differences between these NITs, although there was an underestimation of fibrosis stage compared to patients with CHC [23]. Zeng et al. developed a score that combines clinical and serum direct and indirect markers of fibrosis (α2-macroglobulin, age, GGT, and hyaluronic acid) to assess the presence of significant fibrosis in 372 HBeAg-positive patients). Using cut-off values of <3.0 or >8.7, the Zeng score had an AUROC of 0.77–0.84 for significant fibrosis (F2-F4) with a sensitivity and specificity of >90%. However, 50–60% of patients had scores between the optimal upper and lower cut-offs [24].

The majority of glycoproteins are derived from hepatocytes and have also been evaluated as markers of liver disease severity and progression. A study that analyzed serum N-glycome profiles of 128 patients with CHB found that specific profiles had a correlation with the presence of significant fibrosis, advanced fibrosis, and early cirrhosis with an AUROC of 0.67, 0.74, and 0.75, respectively [25]. Glycans can reflect the differentiation state of cells and, in the case of hepatitis, are considered to reflect the progression of fibrosis. Recently the Mac2-binding protein glycosylation isomer (M2BPGi) has been evaluated as a marker for fibrosis and a predictor for the development of hepatocellular carcinoma (HCC) in patients with CHB [26,27]. Bui et al. investigated the efficiency of M2BPGi to differentiate fibrosis stages in a cross-sectional study that included 177 patients with CHB, using fibroscan as a gold standard. The cut-off value for significant fibrosis (F ≥ 2) was 0.79 with an AUROC of 0.77, 67.3% sensitivity and 70% specificity, and for diagnosing cirrhosis (F4) was 1.3 with an AUROC of 0.91, 88% sensitivity and 87.4% specificity [28]. Many blood-based biomarkers, such as M2BPGi, are influenced by necroinflammatory activity. This results in variable optimal cut-offs for these biomarkers across different chronic liver disease etiology and reduced levels secondary to biochemical responses following therapy.MicroRNAs (miRNAs) are short, noncoding RNAs involved in the epigenetic regulation of multiple intracellular and extracellular signaling pathways and involved in the posttranscriptional regulation of genes. They have been studied by multiple studies for diagnosis, prognosis, and treatment of viral infections [29]. A study that included 123 treatment naïve patients with CHB, identified a panel of miRNAs differentially regulated between F1-F2 to F3-F4. A panel comprising three miRNAs (miR-29a, miR-143 and miR-223) and platelets had an AUROC 0.94 for differentiating F3–4 from F1–2 [30]. These emerging markers are not routinely available and require further clinical utility validation.

### 2.7. Serum Markers and HBV Infection Phase

The natural history of CHB includes five main phases, (1) HBeAg-positive chronic infection, (2) HBeAg-positive chronic hepatitis, (3) HBeAg-negative chronic infection, (4) HBeAg-negative chronic hepatitis, and (5) HBsAg-negative phase, accounting for HBeAg status, HBV DNA levels, ALT, and liver Inflammation [5]. Indications for antiviral treatment typically require elevated HBV DNA (>2000 IU/mL), elevated ALT, and/or at least moderate necro-inflammatory activity and significant fibrosis. Given that therapeutic decisions for CHB include both viral and inflammatory markers and not just disease severity, a biopsy is infrequently obtained in clinical practice. Even prior to the availability of NITs, this was particularly the case for eAg positive or negative chronic infection (with typically normal range ALT). Few studies have validated NITs for the assessment of fibrosis in patients who are HBeAg negative, and prior studies have reported that these patients are at risk of significant liver injury, even with normal ALT. A retrospective study that included 126 HBeAg-negative patients with CHB that had a normal ALT and detectable HBV-DNA showed that 23% had significant inflammation and 10.8% had significant fibrosis [31].

A retrospective study including 184 HBeAg-negative patients with detectable HBV DNA and normal ALT evaluated the predictive accuracy of gamma-glutamyl transpeptidase-to-platelet ratio (GPR) for significant fibrosis and cirrhosis. This GPR score had an AUROC of 0.72 with a sensitivity of 68% and specificity of 68% to identify ≥F2, and an AUC of 0.95 with a sensitivity of 100% and specificity of 89% to identify F4. This study also noted a lower accuracy of FIB-4 and APRI in comparison to GPR to identify ≥F2 or F4 Further validation for serum NITs is needed in patients with HBeAg positive and negative chronic infection [32].

## 3. Imaging Tests:

### 3.1. Vibration Controlled Transient Elastography

Vibration Controlled Transient elastography (VCTE) (Fibroscan®, Echosens, Paris, France) was initially developed in 2003 for the non-invasive assessment of liver fibrosis using one-dimensional elastography [33]. The technology measures the propagation of mild amplitude, low-frequency shear waves, produced by an ultrasound transducer, through the liver tissue to provide an elastic modulus and corresponding liver stiffness measurement (LSM) [34]. Optimal quality requires at least 10 validated measurements and an interquartile range (IQR, that reflects variations among LSM) <30% of the median value (IQR/LSM < −30%) [35]. VCTE has proven to be highly reproducible compared to liver biopsy with excellent inter and intra-observer agreement [36].

VCTE is a quick, safe, and reproducible procedure that can be performed as a point-of-care test in the clinic and is currently the most widely used and best validated of the imaging elastography techniques for assessment of liver fibrosis. Different probes such as the XL probe are available and have reduced scan failure rates, and poor reliability associated with the prior M probes in patients with obesity. Other important LSM confounders include inflammation, cholestasis, congestion, and food intake. Liver stiffness cut-offs vary with the etiology of chronic liver disease. Acute inflammatory injury, for example, associated with hepatitis B reactivation flare or acute HBV infection, will result in high LSM independently of fibrosis stage LSM results should be interpreted carefully by qualified physicians with careful consideration of all the variables that may influence a valid result [35].

#### 3.1.1. Vibration Controlled Transient Elastography (VCTE) in Inactive CHB Infection

HBeAg-negative chronic HBV infection was previously termed the ‘inactive carrier’ phase and is defined by undetectable or low (<2000 IU/mL) HBV DNA levels and normal range ALT [5]. Prior studies showed a significant difference in LSM between HBeAg-negative infection and HBeAg-negative hepatitis, with higher values in the hepatitis group [37,38,39]. LSM value of >6.5 kPa was able to identify the patients with CHB infection that had moderate fibrosis in 35% of cases [40]. VCTE may be useful for the follow-up of the eAg-negative infection. In a study by Wong et al., 316 patients with eAg-negative infection with normal ALT, HBV DNA < 20,000 IU/mL, and non-advanced fibrosis underwent LSM twice with an interval of 44 ± 7 months. Liver fibrosis progression was observed only in 2.8% based on the liver stiffness measurement by transient elastography. Liver biopsy was not performed in this study, but follow-up VCTE after 3 years did not show fibrosis progression, although a significant proportion of these eAg-negative “inactive carriers” with fluctuating HBV DNA developed treatment indications during follow-up [41]. In a recent study from Italy, researchers evaluated the accuracy of non-invasive methods for detecting liver fibrosis in 125 CHB “inactive carriers”. They compared common fibrosis scores such as APRI, Forn’s Index, and FIB-4 with VCTE. Fibrosis scores were not useful to differentiate between patients with LS < 7.5 kPa and those with LS ≥ 7.5 kPa [42].

#### 3.1.2. VCTE in Chronic Hepatitis B

AASLD guidelines for HBV infection state liver stiffness measurements are more accurate than serum fibrosis panels (APRI or FIB-4) in predicting significant or advanced fibrosis [43]. Liver fibrosis assessment is important in making therapeutic decisions in CHB or to evaluate the need for HCC surveillance. The natural history of CHB is variable and characterized by fluctuations degree of necroinflammation. As with serum-based NITs, the accuracy of fibrosis assessment using liver stiffness may be skewed by significantly elevated ALT levels [44]. VCTE thresholds have been proposed to guide the initiation of antiviral therapy in patients with normal ALT levels [45]. Four meta-analyses have been published with varying cut-off values for significant fibrosis and cirrhosis with different sensitivity and specificity (Table 2) [46,47,48,49]. The available studies show that VCTE is better at excluding cirrhosis (F4) than diagnosing significant fibrosis (F2–4). There is significant variation in selecting optimal VCTE thresholds. Based on the metanalysis by Qi X. et al., the optimal cut-off values for F2–4 and F4 were 7.25 kPa and 12.4 kPa, respectively. Previous studies reported varying cut-off values for F2–4 (5.2 to 10.3 kPa) and F4 (9 to 18.2 kPa). There are some limitations of this meta-analysis, including significant heterogeneity among the studies and an inability to calculate VCTE values to account for ALT elevations. However, a previous study looking at the ALT-adjusted cut-off value did not improve the performance of VCTE [50].

### 3.2. Acoustic Radiation Force Impulse (ARFI) Elastography

This imaging method assesses liver stiffness based on regional tissue compression by a short-duration acoustic pulse to measure shear wave propagation using a dedicated US probe and provide an ARFI-shear wave velocity (ARFI-SWV) in m/s. ARFI can be included in commercial B-mode US equipment, making it a readily available technique at imaging centers for fibrosis assessment. Limited studies are available in the literature on assessing the fibrosis stage in CHB. A cross-sectional study using ARFI in 114 patients with CHB for predicting fibrosis showed AUROC of 0.97 and 0.98 with a cut-off value of 1.37 m/s and 1.70 m/s for detecting significant fibrosis and cirrhosis, respectively [51]. A meta-analysis of 21 articles with 2691 patients with CHB and CHC showed ARFI elastography performed well in detecting higher-stage liver fibrosis (F ≥ 3). The AUROC of detecting F ≥ 3 and F ≥ 4 was 0.94 and 0.91, respectively [52]. In a study from Taiwan that included 559 patients with CHN, ARFI showed a good correlation with FIB-4 (r = 0.55) and VCTE (r = 0.69). In this study, serial ARFI measurements were obtained to show its effectiveness in demonstrating reduced “liver stiffness” following antiviral therapy. Among patients for whom serial ARFI measurements were available, in the nontreatment group (*n* = 189), the ARFI value remained unchanged (from 1.11 to 1.11 m/sec; time trend *p* = 0.911), whereas, in the treatment group (*n* = 125), ARFI values declined significantly (from 2.15 to 1.75 m/sec; time trend *p* < 0.001) [53]. Limitations of ARFI include the requirement of a US-trained technician, a narrow range of measured units (0.5–4.4 m/s), technical considerations regarding the selection of the region of interest, co-morbid factors (heart failure, extrahepatic cholestasis, breathing cycle, acute inflammation, post-prandial state, etc.), and inability to directly compare with other SWE or VCTE “stiffness” values [8].

### 3.3. Magnetic Resonance Elastography

Magnetic resonance elastography MRE is an MRI-based technique for the assessment of fibrosis in patients with chronic liver disease (CLD). MRE has been increasingly available in tertiary clinical research centers over the past 15 years [54]. MRE has been increasingly validated as an alternative to liver biopsy for fibrosis staging in MASLD clinical trials [55]. A small study of 63 patients with CHB showed MRE had better performance for significant fibrosis and cirrhosis determined by biopsy compared to simple blood-based markers such as APRI or AST/ALT ratio [56]. In another biopsy-proven cohort of 63 patients with CHB, MRE performed significantly better compared with diffusion-weighted imaging (DWI) for the detection of significant fibrosis (≥F2), advanced fibrosis (≥F3), and cirrhosis (F4) [57]. Ichikawa et al. evaluated the reliability and validity of MRE in diagnosing fibrosis stages in CHB. In this study, MRE demonstrated a significantly better ability to stage biopsy-proven liver fibrosis compared to serum fibrosis markers in 73 patients with CHB. Additionally, interobserver agreement for MRE measured by two observers was excellent [58].

### 3.4. Two-Dimensional (2D) Shear-Wave Elastography (SWE)

2D SWE is an ultrasound-based (US) technique for non-invasive assessment of liver fibrosis, which is embedded in the US machines and allows the interrogation of the tissue by acoustic radiation force impulses into the tissues by focused ultrasonic beams and captures the propagation of resulting shear waves in real-time. Elasticity is displayed using a color-coded image superimposed on a B-mode image, and at the same time, a quantitative estimation of liver stiffness (LS) can be performed in a certain region of interest (ROI) [59]. In an individual patient data-based meta-analysis by Herrmann et al., 2D-SWE was compared with liver biopsy in 400 patients with CHB. The AUROC of 2D-SWE for patients with CHB was 0.91 for diagnosing significant fibrosis and 0.95 for diagnosing cirrhosis [60]. 2D-SWE was compared with TE for assessing liver fibrosis in a study by Zeng et al., with a good correlation between the two imaging tests. In 257 patients with CHB that had histologic diagnosis, Spearman’s rank coefficients were 0.52 for stage F0 (*p* < 0.001), 0.68 for stage F1 (*p* < 0.001), 0.78 for stage F2 (*p* < 0.001), 0.67 for stage F3 (*p* < 0.001), and 0.75 for stage F4 (*p* < 0.001). The AUROC of the 2D-SWE and VCTE for F2–4, F3–4, and F4 were 0.88–0.93 and 0.85–0.91 for 2D-SWE and VCTE, respectively, with no significant difference between these imaging tests [61]. In a metanalysis by Dong et al. of 72 studies, 2D-SWE and MRE for the fibrosis assessment had better diagnostic performance than serum biomarkers, with AUROC of 0.89 and 0.97, 0.95 and 0.97, and 0.94 and 0.97 for significant fibrosis, advanced fibrosis, and cirrhosis, respectively; AUROCs for APRI and FIB-4 to detect significant fibrosis, advanced fibrosis, and cirrhosis were 0.76 and 0.75, 0.74 and 0.77, and 0.77 and 0.82, respectively [62]. In a meta-analysis of 11 studies with 2623 patients with CHB, 2D-SWE with a mean threshold of 7.91 kPa showed a sensitivity of 88%, specificity of 83%, and AUROC 0.92 for detecting significant fibrosis [63].

### 3.5. Combination of Non-Invasive Tests for Fibrosis Assessment

The age-male-albumin-bilirubin-platelets score (aMAP) score was originally developed to predict the HCC risk in liver diseases. A recent study with 2053 patients with CHB, including 889 patients with paired biopsy, evaluated the performance of aMAP for diagnosing liver fibrosis in patients with CHB that were with or without treatment. In the cross-sectional analysis of 2053 patients, the AUROC of aMAP in diagnosing advanced fibrosis (F3–F4) and cirrhosis (F4) were 0.76 and 0.79, respectively, and comparable with those for FIB-4 and APRI. Combining aMAP with LSM (aMAP-LSM model) by calculating aMAP and LSM results before and after treatment, had good performance in diagnosing advanced fibrosis and cirrhosis after treatment with AUROC 0.84 for both advanced fibrosis and cirrhosis compared with LSM alone [64].

## 4. Longitudinal Assessment of Fibrosis Stage with Antiviral Therapy

In chronic liver disease, there is an absence of NITs that can accurately reflect progression or regression in the fibrosis stage, either as part of natural history or following therapy for an underlying etiology [8]. Antiviral therapy in CHB results in viral suppression, fibrosis regression, and reversal of cirrhosis. An important study evaluated the diagnostic utility of APRI and FIB-4 for fibrosis assessment from 575 patients with CHB enrolled in two phase III clinical trials. At baseline, 81–89% of patients with advanced fibrosis or cirrhosis were missed by APRI and FIB-4, and 71% of the patients with mild fibrosis were misclassified as having significant fibrosis. Paired biopsy was available in 298 patients at week 240 of antiviral therapy. There was no correlation between change in fibrosis and APRI or FIB-4, and the majority of patients with persistent advanced fibrosis on antiviral therapy would have been misclassified [65]. Available serum biomarkers include transaminases or acute phase reactants that are likely to show biochemical responses following antiviral therapy in HBV, thus resulting in false negative tests for assessing moderate-advanced fibrosis.

VCTE has been proposed for predicting treatment response after long-term antiviral therapy. In a study of 148 patients, 96 weeks of entecavir therapy was associated with a significant decrease in LSM (from 9.32 ± 3.84 kPa at baseline to 5.41 ± 1.42 kPa after therapy). This change was predictive of histologic improvement in the fibrosis stage with a sensitivity of 74.3% and specificity of 68.8%, and AUROC of 0.70 [66]. In a prospective study with 48 treatment naïve patients, a significant decline in LSM value was observed after 96 weeks of tenofovir disoproxil fumarate (TDF) therapy. Thirty-six patients were followed for 144 weeks, and the median LS value decreased from 13.8 kPa at baseline to 6.4 kPa at week 144, although liver biopsy was not performed in this study [67]. However, in a prospective study by Dong XQ et al., regression in the LSM value was not indicative of liver biopsy-based fibrosis improvement after 78 weeks of treatment with antiviral. In this study, 556 treatment naïve patients underwent paired biopsy at baseline and at 78 weeks. Increased LSM was associated with histologic activity index (HAI). Liver stiffness improvement was associated with improvement in HAI at week 78 (from 11.3 (7.8–16.7) kPa at baseline to 6.4 (5.1–8.8) at week 78) [68].

## 5. Role of Serum Fibrosis Markers and Imaging Tests for Prognosis/Liver Outcomes

NITs have been used to predict the development of HCC and liver decompensation in patients with CHB. In a 5-year follow-up study including 600 patients, NITs, including liver stiffness, FibroTest, APRI, and FIB-4, were assessed for predicting liver-related outcomes. In this study, FibroTest and liver stiffness had the highest hazard ratio with survival. VCTE > 9 kPa and FibroTest > 0.73 were associated with reduced survival [69]. FIB-4 index was able to predict the cirrhosis risk and liver-related complications in 2075 patients with treatment naïve non-cirrhosis CHB. FIB-4 index of >1.29 was associated with an increased risk of cirrhosis and liver-related outcomes in this 15-year follow-up study [70]. In a study from Korea (*n* = 9300), diagnosis of cirrhosis with the combined use of ultrasound and VCTE predicted the development of HCC and liver-related events (LREs) in patients with CHB. TE cut-off value of ≥13 kPa was considered for the diagnosis of cirrhosis. During a median follow-up of 60 months, 5.2% developed HCC, and 8.2% developed LREs. The highest cumulative incidence rate was in the group diagnosed with cirrhosis based on both VCTE and US [71]. The LSM measurement with VCTE at baseline and age was the independent predictor of the LRE development in patients receiving entecavir therapy. In this study, the cut-off LS value of 12.0 kPa had a sensitivity of 93.3%, specificity of 42.2%, and AUROC of 0.74 [72]. Another study with 128 treatment naïve patients with CHB from Korea revealed that the optimal cut-off value for LSM was 19 kPa with a sensitivity of 61.1% and a specificity of 86.2%. Together with age, LSM was an independent predictor for the development of LREs (hazard ratio 1.04; 95% confidence interval 1.00–1.08) [73]. HBV remains the major risk factor for the development of HCC in patients with both treated and untreated CHB. Different variables have been used in the studies to develop models to predict the development of HCC. The presence of cirrhosis is the most important predictive factor in most of the prediction models. Cirrhosis diagnoses in these studies were based on clinical or imaging evidence, which does not differentiate between advanced fibrosis and cirrhosis. In the predictive model studies, liver stiffness measurements by FS have been used as categorical and non-categorical values. The LS value is a significant predictor of the development of HCC. In a study from Korea, 540 patients with CHB were followed up for a median period of 54.1 months, and it was found that there was a significantly reduced risk of HCC development in the sub-cirrhotic range LS value (≤13 kPa) compared to the cirrhotic range (*p* < 0.05) [74]. Various scores like PAGE-B (baseline patient age, gender, and platelets) and Modified REACH-B (risk estimate for hepatocellular carcinoma in chronic hepatitis B) have been developed with different efficacies for the prediction of HCC [75,76]. 2D-SWE has been shown to be predictive of liver-related events in patients with CHB. In a study by Wu et al., 430 patients were followed up for 4 years, and 29 patients developed liver-related events. Multivariate analysis showed liver stiffness measurement by 2D-SWE, spleen longitudinal diameter on US, age, and albumin level were predictive of the liver-related events [77]. The APS score (Age, Platelet, and 2D Shear-Wave Elastography) included age, platelet count, and baseline liver stiffness measurement by 2D-SWE. Based on this score, a cut-off of 60 showed the best discrimination factor for the development of HCC, with AUROC of 0.89, which was better than the Chinese University HCC prediction score (CU-HCC score) and GAG-HCC score (guide with age, gender, HBV DNA level, core promoter mutations, and cirrhosis) [78]. These scores (REACH-B, GAG-HCC, CU-HCC) were developed in Asian cohorts and may have lower performance in non-Asian cohorts. Other scores (mPAGE that include platelet, age, and gender) appear to have better predictive utility in both Asian and Non-Asian cohorts [79].

## 6. Summary

Assessment of liver disease severity is important for treatment decisions and prognosis in CHB, which is characterized by variable periods of necroinflammation and fibrogenesis. In recent years, there has been increased availability and use of non-invasive tests for fibrosis. However, current NITs are not ideal for diagnosing early stages of fibrosis and for discriminating adjacent fibrosis stages. Among the imaging methods, VCTE is increasingly available as a point-of-care test and has been extensively studied in both eAg-negative infection and hepatitis phases. In eAg-negative infection, LM is lower in patients with CHB that haveactive inflammation. LSM can be used to monitor the disease activity along with blood tests. In patients with CHB, VCTE is better at predicting advanced fibrosis with a cut-off value of 7.2 to 8.4 kPa for significant fibrosis and from 10.3 to 13.4 for cirrhosis. In predicting significant or advanced fibrosis, liver stiffness measurements (elastography) are more accurate than serum fibrosis panels (e.g., APRI or FIB-4). Although other elastography techniques have not been as well validated, they have demonstrated promising results in terms of reproducibility and applicability in clinical practice (Table 3). VCTE has clinical utility for the diagnosis of advanced fibrosis and cirrhosis, with better performance than simple markers, but current NITs have limited performance for predicting fibrosis progression or regression. Simple serum-based biomarkers are limited by indeterminate results in a significant proportion of patients but are routinely available, useful in resource-limited clinical practice, and have better diagnostic performance for advanced disease. Algorithms that combine imaging and serum-based biomarkers appear promising for improved predictive accuracy but require further validation. Likewise, there are no validated NIT thresholds for fibrosis assessment in HBV-MASLD or HBV-HCV/ HIV co-infection.

## 7. Future Directions

Although ‘omics-based analytic discovery tools have resulted in several blood-based candidate biomarkers for “liquid biopsy”, translating these to clinical practice remains challenging. Prospective validation of candidate biomarkers for diagnostic and prognostic use may take several years. Furthermore, due to the complex interactions between host genetics, virus, and environmental factors that influence variable natural history of CHB disease severity, developing reliable molecular and genetic signatures for early diagnosis and prognosis in CHB will likely continue to pose a considerable challenge for blood-based biomarker development. Imaging-based techniques such as MRE and corrected T1 mapping continue to be developed for fibrosis severity and inflammation for MASLD but have not yet been validated for assessing disease severity in patients with CHB. Overcoming the diagnostic limitations of liver biopsy as the reference standard for fibrosis and necroinflammation still remains a challenge in biomarker development, but emerging tools such as in vivo bioimaging and digital AI mapping of histology could provide alternative options in the future. However, given that the majority of patients with CHB live in resource-limited areas, the pragmatic application of these next-generation imaging and blood-based biomarkers appears very limited.

## Figures and Tables

**Table 1:** Non-invasive serum tests’ performance in detecting fibrosis in patients with chronic hepatitis B.

Biomarkers	Components	Formula	Benefits	Limitations	Cost	False Positivity	Thresholds
**APRI**	AST platelets	[(AST (IU/L)/(AST Upper Limit of Normal in IU/L)/(Platelets in 10^9^/L)] × 100	Simple biomarkers Accessible	Limited accuracy to for significant fibrosis	Low	Age, immune thrombocytopenia,	<0.5 (F0–1) and >1.5 (F2–4) <1 (F0–3) >2 (f4)
**FIB-4**	Age AST Platelets ALT	[age (years) × AST (IU/L)]/[platelet count (10^9^/L) × √ALT (IU/L)]	Simple biomarkers Accessible	Limited accuracy for advanced fibrosis	Low	Immune thrombocytopenia, age.	<1.45 (F0–2) and >3.25 (F3–4)
**FibroTest**	Alpha-2macroglobulin Apolipoprotein A1 Haptoglobin GGT Bilirubin	Patented	Accessible	Includes indirect biomarkers that can be influenced by other causes of inflammation	High	Haemolysis, Gilbert’s disease, cholestasis, immune thrombocytopenia, inflammation, age, exercise, non-fasting	>0.58 for advanced fibrosis >0.74 for cirrhosis No specific cut-offs for CHB
**Forns Index**	Age, GGT, cholesterol, and platelets	=7.811 − 3.131 × ln platelet + 0.781 × ln GGT + 3.647 × ln age − 0.014 × cholesterol	Simple biomarkers Accessible	Needs more validation	Low	Thrombocytopenia, inflammation, age, non-fasting	≥4.05 to rule-in significant fibrosis
**Hui Score**	BMI, Bilirubin, Albumin and platelets	PP = exp (3.148 + 0.167 × BMI + 0.088 × bilirubin[μM] − 0.151 × albumin[g/l] − 0.019 × platelet [109/l])/(1 + exp(3.148 + 0.167 × BMI + 0.088 × bilirubin[μM] − 0.151 × albumin[g/l] − 0.019 × platelet[109/l]))	Simple biomarkers Accessible	Needs more validation	Low	Haemolysis, Gilbert’s disease, cholestasis, immune thrombocytopenia, inflammation, age, exercise, non-fasting	≤0.15 to rule-out significant fibrosis

AST, aspartate transaminase; APRI, AST platelet ratio index; IU/L, International unit/Liter; FIB-4, Fibrosis-4; ALT, Alanine transaminase; GGT; gamma glutamyl transaminase; BMI, Body Mass Index.

**Table 2:** Summary of Systematic review and meta-analysis studies for the diagnosis of biopsy-determined fibrosis stage using VCTE in patients with chronic hepatitis B.

Author (Ref.) (Year)	Fibrosis, No of Studies, (No of Patients)	Prevalence	Optimal Cut Off (kPa)	Sensitivity	Specificity	Diagnostic Odd Ratio (CI)	AUROC (CI)
**Qi X et al.** [46] **(2018)**	F2–4: 35 (*n* = 6202)	--	7.25 (5.2–10.3)	0.78 (0.73–0.81)	0.81 (0.77–0.84)	14.44 (10.80–19.30)	0.86 (0.83–0.89)
	F4: 41 (*n* = 7205)	--	12.4 (range 9–18.2)	0.84 (0.80–0.88)	0.87 (0.84–0.90)	36.63 (25.38–52.87)	0.92 (0.90–0.94)
**Li Y et al.** [47] **(2016)**	F ≥ 2: 27 (*n* = 4386)	--	7.2 (5.8–8.8)	0.81 (0.76–0.85)	0.82 (0.76–0.87)	--	0.88 (0.85–0.91)
	F ≥ 3: 27 (*n* = 4386)	--	9.1 (7.0–13.5)	0.819 (0.748–0.874)	0.87(0.82–0.90)	---	0.91(0.88–0.93)
	F4: 27 (*n* = 4386)	--	12.2 (9.0–16.9)	0.86(0.82–0.90)	0.88(0.84–0.90)	--	0.93(0.91–0.95)
**Xu X et al.** [48] **(2015)**	F2–4: 14 (*n* = 2318)	51.8% (range 15–83%)	--	Europe: 73% (69–77%) Asia: 73% (69–76%)	Europe: 66% (62–70%) Asia: 82% (79–85%)	11.19 (6.63–18.89)	0.823(SE = 0.02)
	F4: 18 (*n* = 2996)	17.6% (range 4–52%)	--	Europe: 67% (57–76%) Asia: 81% (77–85%)	Europe: 92% (89–93%) Asia: 86% (85–88%)	26.87 (17.88–40.38)	0.91 (SE = 0.01)
**Chon Y E et al.** [49] **(2012)**	F2:18 (2772)	--	7.9 (6.1–11.8)	74.3%	78.3%	--	0.86 (0.86–0.86)
	F3:18 (2772)	--	8.8 (8.1–9.7)	74.0%	63.8%	--	0.89 (0.89–0.89)
	F4:18 (2772)	--	11.7 (7.3–17.5)	84.6%	81.5%	--	0.93(0.93–0.93)

AUROC, Area under receiver operating curve, CI, confidence Interval, SE, standard error.

**Table 3:** Imaging tests’ performance in detecting significant fibrosis and cirrhosis in patients with chronic hepatitis B.

	Technical Limitations	Performance for Intermediate Fibrosis Stage	Cost and Availability	Confounding Factors and False Results	Failure	Cut-Off	Follow up of Dynamic Fibrosis Changes
**Transient elastography (VCTE)**	Require training and experience, No B-mode image, unable to select liver region of interest	Overlapping LSM range	Not widely available particularly in the resource limited area	Acute hepatitis, inflammation, Non-fasting, intense exercise, hepatic venous congestion, inflammation,	Depending on the operator experience, Narrow intercostal space, ascites, body habitus	Significant Fibrosis: 7.25 kPa Cirrhosis 12.4 kPa in CHB infection	limited data
**ARFI Elastography**	Can be included in the standard B mode US equipment	No data	Required trained technician, Expensive, Narrow range of values	acute hepatitis, liver inflammation, transaminitis flares, obstructive cholestasis, hepatic congestion, and infiltrative liver diseases non-fasting, intense exercise, anatomical and physiological variation (Left vs Right lobe, breathing cycle)		Significant Fibrosis: 1.34 m/s Severe fibrosis: 1.55 m/s Cirrhosis: 1.8 m/s	No data
**2D Shear wave elastography**	Require dedicated US training	No data	Increasingly available	acute hepatitis, hepatic inflammation or infiltration, non-fasting, exercise, right heart failure, extrahepatic cholestasis, breathing cycle (end-expiration vs. end- inspiration)	Higher failure rates than serum tests: BMI, tissue depth > 2–3 cm below skin surface	No data	Limited data
**MRE**	Requires specializes radiologist and technician	No data	Highly expensive, Not available outside specialized imaging centers	Inflammation, cholestasis, hepatic venous congestion, postprandial state, and right heart failure	Higher failure than serum tests: waist circumference/BMI, claustrophobia, iron deposition, massive ascites, higher field strength (3 T vs. 1.5 T)	No data	No data

VCTE, vibration-controlled transient elastography; LSM, liver stiffness measurement; CHB, Chronic Hepatitis B; 2D, Two dimensional; ARFI, Acoustic Radiation Force Elastography; US, Ultrasound, BMI, Body Mass Index; MRE, Magnetic Resonance Elastography.

## Data Availability

Not applicable.
